# Cardiac Implantable Electronic Devices in Different Anatomical Types of Persistent Left Superior Vena Cava: Case Series and Brief Review of the Literature

**DOI:** 10.3390/diagnostics12112596

**Published:** 2022-10-26

**Authors:** Cosmin Gabriel Adavidoaei, Ana Maria Haba, Irina Iuliana Costache, Viviana Onofrei, Cristian Mihai Stefan Haba, Ciprian Rezus, Andreea-Maria Ursaru, Nicolae Dan Tesloianu

**Affiliations:** 1 Department of Cardiology, Emergency Clinical Hospital “Sf. Spiridon”, 700111 Iasi, Romania; 2 Department of Internal Medicine, “Grigore.T. Popa” University of Medicine and Pharmacy, 700115 Iasi, Romania; 3 Department of Internal Medicine, Emergency Clinical Hospital “Sf. Spiridon”, 700111 Iasi, Romania

**Keywords:** persistent left superior vena cava, pacemaker, defibrillator, coronary sinus, congenital anomaly

## Abstract

Persistent left superior vena cava (PLSVC) is the most common congenital malformation of the thoracic venous system, being present in 0.3% to 0.5% of the general population. In the majority of the cases, PLSVC is asymptomatic, but in certain patients, it can manifest through several symptoms, such as arrhythmias and cyanosis, especially when it is associated with complex cardiac pathologies. The clinical significance of this venous anomaly depends on the anatomical variant of the drainage site. In this article, we will present the experience of our clinic, with patients with PLSVC that were diagnosed intraprocedurally, during cardiac pacemaker (CP) or cardioverter defibrillator (ICD) implantation, highlighting the technical difficulties that this anomaly poses for cardiac device implantation. Out of 4000 patients who were admitted to our clinic for CP or ICD implantation, we encountered six cases of PLSVC (four reported in this article and two previously published) corresponding to different anatomical types of this congenital anomaly. In all of these situations, we had to adapt our technique to the patient’s anatomy in order to avoid certain complications, the most serious being the improper placement of the right ventricle lead at the level of the coronary sinus.

## 1. Introduction

The congenital anomalies of the thoracic venous system represent a rare medical entity, mostly asymptomatic, that can become clinically manifest in the context of other cardiovascular anomalies (heart structure anomalies, arterial anomalies) and can pose problems to physicians when medical procedures are undergone at the level of the thoracic veins. Persistent left superior vena cava (PLSVC) is the subject of our discussion, and it belongs to the systemic congenital anomalies [1]. Described for the first time in 1950 by Edwards and Dushane, the real incidence of this anomaly is difficult to assess due to its predominantly asymptomatic nature, being estimated at approximately 0.5% in the general population [2]; however, the real incidence seems to be higher due to the continuous increase in the number of procedures involving the central venous approach, which lead to the incidental finding of this condition. The early diagnosis, preferably preoperative, of persistent left superior vena cava is important in the context of cardiac catheterization, the implantation of transvenous cardiac devices and cardiovascular surgery, since any change in the normal anatomy can lead to the approach of new techniques and the choice of another operative strategy. In the field of cardiac pacemakers (CP) and implantable cardioverter defibrillators (ICD), access to the right heart can be difficult, possibly leading to the incorrect placement of the pacing lead [3]. 

In this article, we share the experience of our team regarding the PLSVC patients incidentally diagnosed during CIED procedures by emphasizing the encountered difficulties and our approach for each of these cases.

## 2. Case Reports

**Figure 1:**
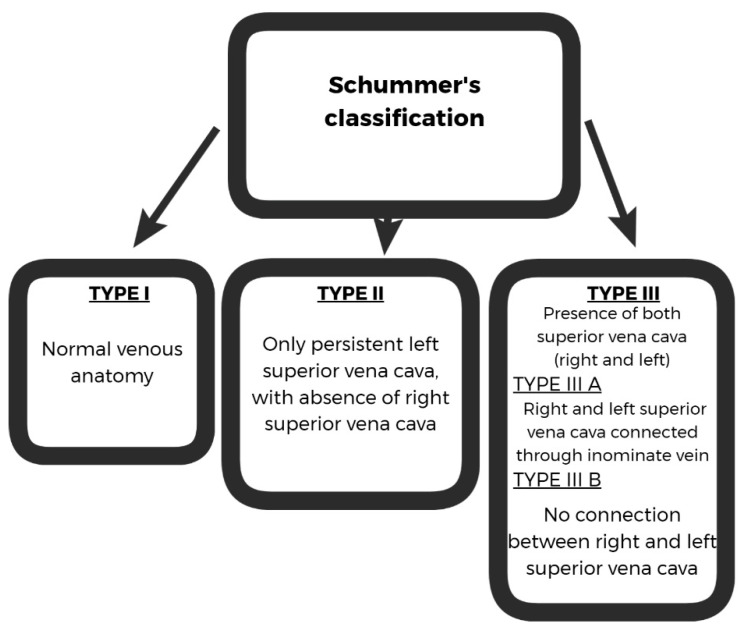
Schummer’s classification of persistent left superior vena cava.

Several classifications for PLSVC are reported in literature. The most frequently used is Schummer’s classification [4] of the supracardial venous system, as shown in Figure 1.

To date, in our clinic, during different device implantations, we encountered venous anomalies that correspond to most types of persistent left superior vena cava according to this classification. At present, over 4000 patients require implantation of cardiac devices (pacemakers, implantable defibrillators, or cardiac resynchronization therapy) in our clinic. Among these cases, we identified six patients who had PLSVC, which leads to an incidence of at least 0.15% of this anomaly in the group of patients who required implantable devices in our clinic. It should be mentioned that, in these cases, we met several types of this congenital anomaly (two cases of type II, two cases of type III A, and two cases of type III B according to Schummer’s classification); however, in this article, we will present only four cases, since two of them have already been published [5,6]. None of these patients met the criteria for cardiac resynchronization therapy.

### 2.1. Case 1. (Type IIIB)

**Figure 2:**
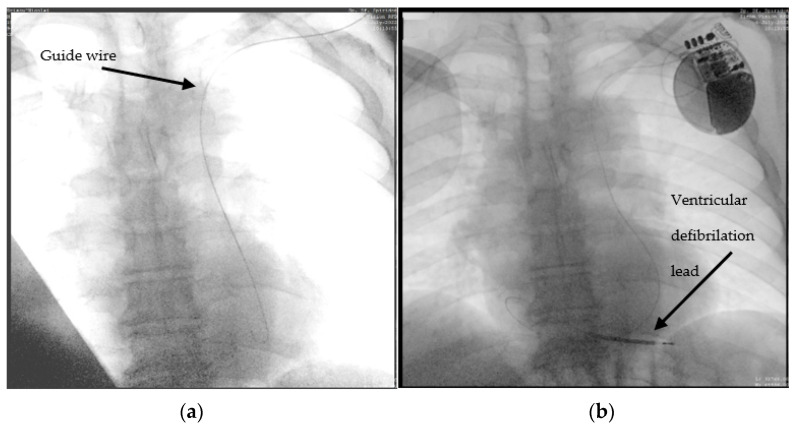
Advance of the ICD ventricular lead through persistent left superior vena cava, coronary sinus, right atrium, and finally, the right ventricle: (**a**) the guide wire (marked) descending to the left side of the spine; (**b**) the ventricular lead placed through the left SVC and coronary sinus into the RV.

A 63-year-old male patient, with multiple cardiovascular pathologies (an anteroseptal myocardial infarction for which a balloon percutaneous transluminal angioplasty was performed) sent to the emergency unit for the occurrence of fast-paced palpitations, associated with anterior chest pain. The initial electrocardiogram objectified ventricular tachycardia, for which one external electrical shock was delivered (150J) coinciding with the restoration of sinus rhythm. The blood tests revealed a normal hemogram, elevated Nt-proBNP, and positive myocardial cytolysis enzymes. Considering the anamnesis and paraclinical data, a coronary angiography was performed, which excluded the possibility of an acute myocardial infarction. According to current guidelines [7], the next step was the implantation of an ICD. During ICD implantation, puncture of the left subclavian vein, revealed PLSVC, incidentally found during the advancement of the guide wire from the left subclavian vein to the superior vena cava (SVC) (Figure 2a). A venography was performed, which showed no connection between right and left superior vena cava (type IIIb). With some difficulty, we managed to advance with a 9F defibrillation lead (Boston Scientific RELIANCE 4-FRONT™, length 64 cm) through the coronary sinus in the right atrium, and then using a J-shaped stylet to the right ventricle. Finally, we placed the lead at the right ventricular apex, as can be seen in Figure 2b.

### 2.2. Case 2. (Type IIIa)

**Figure 3:**
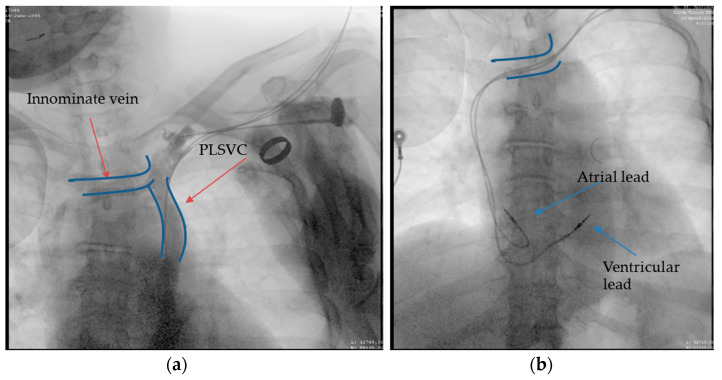
Atrial and ventricular lead placement through innominate vein, right superior vena cava, right atrium, and ventricle: (**a**) the contrast injection in the left subclavian vein showing the presence of PLSVC and innominate vein, which ensures communication with right superior vena cava; (**b**) the final result after we placed the leads at the level of the right atrium (RAA) and right ventricular septum approaching the innominate vein and avoiding the coronary sinus.

**Figure 4:**
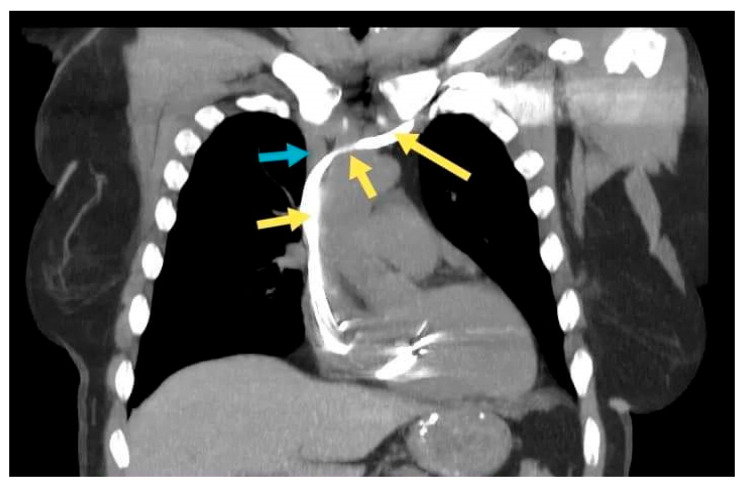
Coronal section. The yellow arrows: the lead in the subclavian vein, persistent left superior vena cava, the innominate vein, and the right superior vena cava. The blue arrow: the right brachiocephalic vein.

A 66-year-old female patient diagnosed with sick sinus syndrome (tachycardia–bradycardia syndrome: sinus bradycardia alternating with paroxysmal atrial fibrillation (AF)), based on 24 h ECG Holter monitoring, symptomatic through repetitive syncope, fatigue, and palpitations, was referred to our clinic for the implantation of a pacemaker. During the dual-chamber pacemaker implantation, the abnormal route of the guide wires was noticed, and we decided to perform a venography through the subclavian vein already punctured (Figure 3a). This not only confirmed the presence of persistent left superior vena cava, but also a communication with the right superior vena cava through a small vein called the innominate vein (type IIIa). Faced with this situation, we decided to approach this small caliber vein to facilitate the implantation of the leads without passing through the coronary sinus. First of all, we managed to insert the guide wires into this vein using rotational movements, and for the leads we used a straight stylet. After reaching the atrium, we passed the tricuspid valve with a specific stylet, and the lead was placed at the right ventricular septum. Finally, the second lead was placed in the right atrial appendage (RAA) (Figure 3b). A thoracic angio-CT was performed, to better characterize the venous anomaly (Figure 4). 

### 2.3. Case 3. (Type II)

**Figure 5:**
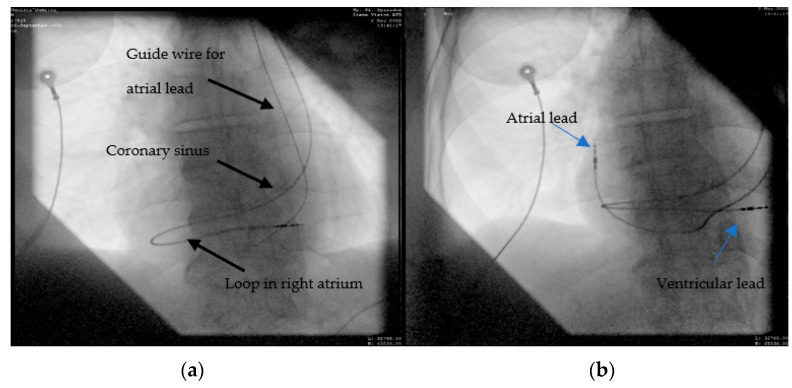
Atrial and ventricular lead placement through PLSVC and coronary sinus: (**a**) the ventricular lead crossing a large coronary sinus and leaving behind a significant loop in the right atrium; (**b**) the final result with the leads at the level of the right atrium and right ventricular apex.

A 69-year-old male patient, diagnosed with sick sinus syndrome (tachycardia–bradycardia syndrome: sinus bradycardia alternating with paroxysmal atrial fibrillation (AF)), based on 24 h ECG Holter monitoring, symptomatic through syncope, was referred to our clinic for the implantation of a pacemaker. According to the current guidelines [8], the patient had an indication of a dual-chamber pacemaker. During this procedure, puncture of the left subclavian vein revealed as in other cases, an abnormal route of the guide wire. At that time, after a venography was performed, we knew that we are again dealing with another case of persistent left superior vena cava, but only post-procedurally; when thoracoabdominal angio-CT was performed, we had the confirmation that this was a case of type II PLSVC, which involves the embryological involution of right superior vena cava. Furthermore, during this exploration, it was found that this patient had multiple venous anatomical anomalies: right renal artery with two branches emerging from the aorta, left renal vein anterior, and posterior to the aorta (annular shape). The suspicion of its presence was raised pre-procedurally due to the increased size of the coronary sinus during the echocardiographic examination, but we did not have confirmation at that time. Just as in the first case, we adopted the same technique for the ventricular lead. Using a straight stylet, we passed through the coronary sinus, and then, with a J-shaped stylet, through the tricuspid valve in the right ventricle, leaving behind a significant loop of the lead at the level of the right atrium (Figure 5a). Finally, using the same route, the second lead was placed in the right atrial appendage (Figure 5b).

### 2.4. Case 4. (Type IIIB)

**Figure 6:**
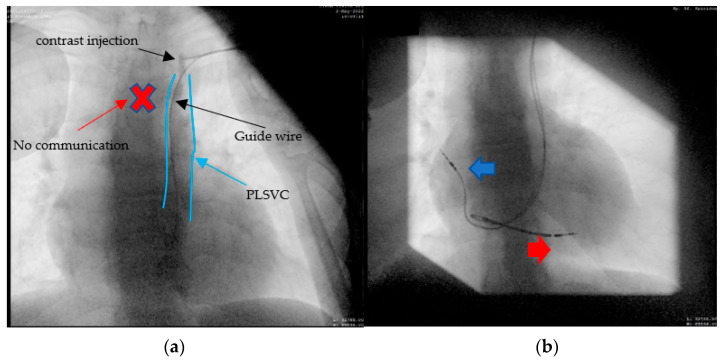
(**a**) The passage of the guide wire to the left side of the spine and then the venography confirming the presence of PLSVC, no communication with the right vena cava can be seen; (**b**) the defibrillation leads can be seen on the trajectory of the left superior vena cava (LSVC), through the coronary sinus (CS) into the right ventricular apex (RV)—red arrow. The atrial lead can be seen through the LSVC, coronary sinus, and right ventricle in the right atrial appendage (RA)—blue arrow.

A 60-year-old female patient, with multiple cardiovascular risk factors (diabetes, dyslipidemia, hypertension), diagnosed with dilated cardiomyopathy with an ejection fraction of 15% and LV diameter of 64 mm 6 months ago, under maximal treatment for heart failure, was referred to our center after being diagnosed with sick sinus syndrome (tachycardia–bradycardia syndrome: sinus bradycardia alternating with paroxysmal atrial flutter) based on 24 h Holter ECG monitoring, symptomatic through syncope. A coronary angiography was performed, revealing normal epicardial coronary arteries, thus excluding an ischemic cause for the dilated cardiomyopathy. According to the current guidelines [9] in the primary prevention of sudden cardiac death, the next step was the implantation of a bicameral implantable cardioverter defibrillator (ICD). Left subclavian access was performed, but fluoroscopic evaluation again revealed an abnormal trajectory of the guide wire, raising the suspicion of a venous anomaly. Same as in case 2, the venography was performed through the subclavian vein, which evoked a persistent left superior vena cava, without any communication with the right superior vena cava (Figure 6a). Post-procedurally thoracoabdominal angio-CT revealed the presence of the right superior vena cava (type IIIb). This time, a 9F defibrillation lead (Medtronic Spring Quattro™, length 62 cm) was advanced through the coronary sinus in the right atrium, and again, using a J-shaped stylet, to the right ventricle, where it was placed at the apical level. In this case, we encountered some difficulties as we advanced the lead, which we passed by slightly retracting the stylet and leaving the tip of the probe free. After that, we placed the atrial lead at the right atrium level (Figure 6b).

## 3. Discussion

Persistent left superior vena cava is a rare anatomic condition; however, it remains the most common of the thoracic venous system, with an incidence of 0.5% in the general population. Excluding the associated comorbidities or cardiac anomalies, PLSVC is asymptomatic, most of the cases being diagnosed incidentally during the implantation of different devices at the level of the thoracic veins. Two types of PLSVC have been reported in the literature. In the majority of the cases, PLSVC connects to the right atrium via the coronary sinus, and it represents about 80–90% of the anomalies of the SVC. In the other 10–20%, PLSVC connects to the left atrium [10]. Regarding the anatomical variations, the most common type, representing 90% of PLSVC cases, is the presence of both right and left superior vena cava. In 30% of the cases, a communication between the right and left superior vena cava through a bridging innominate vein was reported. A very rare variation is the presence of PLSVC only, with the absence of the right superior vena cava [11]. Current medical practice is associated with an increasing number of cardiac implantable electronic device (CIED) procedures, which has revealed a higher incidence of PLSVC than originally estimated. Furthermore, PLSVC causes additional difficulty in CIED implantation due to the important alterations of the venous system anatomy [12].

### 3.1. Embryological Development

PLSVC is an anomaly originated during embryological development. A detailed understanding of embryology is essential for both the clinician and the physician implanting a medical device, in order to know the normal anatomical variants and to identify possible congenital anomalies [3]. In the 5th week of intrauterine life, the fetal venous system includes three pairs of veins: vitelline, umbilical, and cardinal. Gradually, the left vitelline vein and the left and right umbilical veins regress, while the right vitelline vein will form the inferior vena cava. The main venous system involved in the origin of persistent left superior vena cava is represented by the superior and inferior vitelline veins, which join on the same side and form the common cardinal veins. There are also two transverse venous plexuses, one superior and one inferior, which ensure communication between the two superior cardinal veins. Starting at the 8th week of intrauterine life, the cranial parts of the superior cardinal veins form the jugular, subclavicular, and brachiocephalic veins on each side, and the caudal part of the right superior cardinal vein, together with the common cardinal vein, forms the right superior vena cava. In normal embryological development, on the left side, the caudal segment initially forms the left superior intercostal vein, then, together with the common segment, it involutes, turning into Marshall’s ligament. If they do not regress, they form the persistent left superior vena cava. There may also be a left brachiocephalic vein (formed by the superior transverse venous plexus) that connects the two superior vena cava and, in this case, bears the name of the innominate vein. In the case of persistent left superior vena cava, the transverse venous plexus can also suffer total regression without further development of the lower venous plexus, thus resulting in the absence of this bridging vein [13].

### 3.2. Diagnosis

The diagnosis of persistent left superior vena cava should be taken into consideration each time a dilated coronary sinus is discovered on transthoracic echocardiography. The most frequently used echocardiographic view is the parasternal long axis, where the coronary sinus appears as a circular structure with a diameter over 1 cm, at the junction between the left atrium and ventricle. However, the coronary sinus can be evaluated in a modified apical four-chamber view, where the lumen at the sinus can be seen opening in the right atrium [10,14]. The gold standard for the diagnosis of PLSVC is venous angiography [14]. The diagnosis can sometimes be difficult, and it is usually conducted incidentally, since hemodynamics in these patients can be normal and clinical symptoms are mostly absent [15]. 

The transthoracic echocardiography plays an important role in the diagnosis of PLSVC. The direct signs are the existence of the duct-like structure and the blood flow spectrum in the left upper part of the chest, and the indirect sign is the dilated coronary sinus [16]. 

One method used to confirm the presence of PLSVC is the bilateral “bubble study”, with the injection of agitated saline into both the left and the right peripheral arm veins [17]. Normally, the bubbles injected into the peripheral arm veins should first reach the right atrium, with subsequent opacification of the right ventricle. Agitated saline bubbles are not seen in the coronary sinus, because they are destroyed during transpulmonary passage. In the presence of PLSVC, the agitated saline bubbles are seen first in the coronary sinus, followed by right heart opacification. In type II PLSVC, the agitated saline bubbles first enter into the dilated coronary sinus from both peripheral arm veins [18].

Different literature reports have presented several techniques to confirm the diagnosis of PLSVC, such as: angiographic examination (venogram) with bolus contrast injection through the venous catheter, two-dimensional transthoracic echocardiography with injection of contrast material from a peripheral left arm vein, transesophageal echocardiography, computed tomography (CT) of the chest, and magnetic resonance imaging [10].

### 3.3. Clinical Implication

In most cases, PLSVC is a benign condition, but in some situations, it can have important clinical implications. It is hard to detect by physical examination, and it is usually noticed accidentally during the process of intravascular invasive procedure, such as pacemaker implantation, cardiac electrophysiological examination, and central venous hemodialysis catheterization [19].

Inserting different devices (pacemaker or defibrillator leads, central venous catheters) may be difficult because of the narrow opening of the coronary sinus to reach the right atrium. Similarly, when placing a central venous catheter into a PLSVC, it may lead to confusion with some other positions, such as the subclavian or carotid artery, or even the pleural space [10]. Cardiac arrhythmias, such as atrial and ventricular fibrillation, have been reported in some patients. Arrhythmias may result from the dilatation of the coronary sinus opening, which causes the stretching of the atrioventricular node and bundle of His, and is more often seen in the cases with only persistent left superior vena cava (type II according to Schummer’s classification) [20]. Furthermore, some studies have shown that PLSVC has significance in the induction and maintenance of atrial fibrillation, in almost 50% of the patients. Pre-radiofrequency ablation computer tomographic examination can be useful for the diagnosis of a possible PLSVC anatomy in patients with atrial fibrillation, in addition to the normal evaluation of pulmonary venous anatomy [21].

PLSVC, through its multiple anatomical and electrical communications with the atria, may generate repetitive rapid discharges with shorter activation cycle lengths, which promotes the initiation and maintenance of atrial fibrillation and sudden death [22]. Congenital diseases, such as atrial septal defect, are commonly associated with PLSVC, and it may predispose patients to cerebrovascular accident, paradoxical systemic air emboli, or arterial thromboemboli. PLSVC can be associated with multiple other congenital anomalies [10]. In our series of cases, we encountered two patients where associated congenital anomalies were identified. In the reported case 3, angio-CT examination found that the patient had multiple venous anatomical anomalies: right renal artery with two branches emerging from the aorta, left renal vein anterior and posterior to the aorta (annular shape). Another associated congenital anomaly, with stronger clinical implications, was described in a previously published case. In this case, the patient was also diagnosed with subvalvular aortic stenosis, determined by a subaortic membrane, which is a rare finding, with only few similar cases being reported in the literature [5]. It has been observed that patients with PLSVC can also present conduction disorders, which arise as a result of the histological abnormalities caused by the dilated coronary sinus [23]. The 10–20% of patients with PLSVC, which drains blood to the left atrium, may have obvious clinical cyanosis due to the left-to-right shunt, and these cases are always associated with congenital heart diseases (ventricular septal defect, atrial septal defect, or other cardiovascular malformations) [24].

During device implantation, the presence of PLSVC should be suspected whenever a guide wire takes a left downward course. In this situation, the right superior vena cava can be present, but is difficult to cannulate because of a sharp angle created with either the subclavian or the innominate vein. The absence of the right superior vena cava cannot be excluded without additional vascular imaging [25]. Transvenous introduction of a lead from the right atrium to the right ventricle, through the coronary sinus, may become a technically demanding procedure in subjects with PLSVC, especially when the bridging innominate vein or right superior vena cava is absent [11].

In our center, left subclavian vein access is usually preferred, based on the experience of the interventional cardiologists. In the reported cases, PLSVC was diagnosed when the guide wire entered via the left subclavian vein, and descended on the left side, instead of crossing the vertebral column. Performing a venography excludes a possible arterial approach and confirms the diagnosis and type of persistent left superior vena cava. In these types of cases, some studies suggest changing the vascular approach through the right side, only in the cases in which right superior vena cava is present. Giving the experience of our interventional cardiology team, and also the fact that all of the patients were right-handed, it was decided to continue on the left side, and the atrial and/or ventricular leads were advanced via persistent left superior vena cava and coronary sinus. Advancement of the right ventricular lead through the tricuspid annulus was technically challenging due to acute angle between coronary sinus ostia and right atrium. In most patients, it was possible to fixate the ventricular lead to the septum, with the exception of some patients in whom, despite multiple attempts and the use of different shapes of the stylets, the septal fixation of the lead was not possible, and it was placed at the level of the apex by shaping of the stylet and forming a loop within the atrium. Additionally, in the case of defibrillators, the lead was placed at the level of the apex, because of its greatest efficacy, and can be accomplished by forming the stylet into a U-shape in the right atrium or even placing the proximal ICD coil into the coronary sinus and the distal lead coil into the right ventricular outflow tract. When considering only persistent left superior vena cava, and with the absence of the right side, an epicardial implantation should be considered. Over time there have been improvements in catheter types and techniques, which has allowed the right placement of atrial and right ventricular leads in dual-chamber pacemakers [12]. In patients with PLSVC who underwent CIED procedures, serious complications have been reported, such as angina, arrhythmias, cardiogenic shock, and even cardiac arrest [26]. The most important complication consists in the incorrect placement of the lead at the level of coronary sinus, which can lead to a lack of pacing capture or to coronary sinus perforation and cardiac tamponade. To avoid this complication, fluoroscopic examination of several incidents is imperative. In our cases, there were no incidents reported. The main concerns for physicians implanting a cardiac device in a patient with PLSVC are achieving the proper lead placement and function, with good parameters, preventing lead dislodgement, and minimizing radiation exposure. All patients diagnosed with PLSVC in our clinic who received a cardiac implantable electronic device presented with adequate lead parameters (sensing, pacing, and impedance) at the time of the implant, and later at 1, 6, and 12 months.

Considering the low incidence of these types of cases, performing routine imaging procedures for the diagnosis of PLSVC is not justified. Before implantation, all patients undergo a transthoracic echocardiography, and if high suspicion of PLSVC (dilated coronary sinus) arises, a peripheral venography is required. In those cases with improper visualization of the thoracic venous system, a thoracic angio-CT will be performed. When we refer to intraprocedural diagnosis of PLSVC by performing venography at the subclavicular level, in those cases when the innominate vein is present, we prefer this approach to avoid passing through the coronary sinus, and thus, a possible inadequate placement of the lead at this level. If this is not possible (e.g., small caliber of the vein), the lead will be placed through the PLSVC in right atrium and ventricle. Since venography does not allow us to differentiate type II from IIIB of PLSVC, we do not recommend changing the approach to the right side.

## 4. Conclusions

PLSVC is a rare venous congenital anomaly, usually asymptomatic, that can become a real challenge when implanting a pacemaker or defibrillator. Through the cases reported in this article, we show that implantation of cardiac devices can be technically feasible. In our case series, we aimed to address the main concerns regarding the proper implantation of CIEDs in patients with PLSVC. The focus points for a successful procedure are proper lead placement and adequate lead stability and device parameters. As a result, the interventional cardiology team should have a good understanding of various venous system anatomies, and adapt using adequate lead types, different stylet shapes, and active lead fixation.

## Data Availability

Not applicable.
